# From the Past to the Future: Natural Sound Recordings and the Preservation of the Bioacoustics Legacy in Portugal

**DOI:** 10.1371/journal.pone.0114303

**Published:** 2014-12-04

**Authors:** Paulo A. M. Marques, Daniel M. Magalhães, Susana F. Pereira, Paulo E. Jorge

**Affiliations:** 1 Unidade Investigação em Eco-Etologia, ISPA-IU, Lisboa, Portugal; 2 Museu Nacional de História Natural e de Ciência, Universidade de Lisboa, Lisboa, Portugal; University of Brasilia, Brazil

## Abstract

The preservation of historical and contemporary data safeguards our scientific legacy. Bioacoustic recordings can have historical as well as scientific value and should be assessed for their conservation requirements. Unpreserved bioacoustics recordings are generally not referenced and are frequently at high risk of loss by material degradation and/or by misplacement. In this study we investigated the preservation status of sets of natural sound recordings made in Portugal from 1983 until 2010 inclusive. We evaluated the recordings on the basis of their rate of loss, the degree to which unpreserved recordings could be preserved, and their risk of loss. Recordists of animal sounds were surveyed (by questionnaire or interview) to identify sets of recordings and to collect information on their quality and state of preservation. Of the 78 recordists identified, we found that 32% of the recordings have an unclear status and that only 9% of the recordings are lost. Of the c. 6 terabytes of unpreserved sound recordings discovered, an estimated 49% were recoverable. Moreover, 95% of the recoverable sets of recordings were at high risk of loss by their being misplaced. These risks can be minimized if recordists are persuaded to deposit their material in an institution committed to long-term curation of such data (e.g. sound archives). Overall, the study identified a considerable body of unpreserved animal sound recordings that could contribute to our scientific heritage and knowledge of the biodiversity found in Portugal. It highlights the need to implement effective policies to promote the deposit of recordings for preservation and to reverse the present scenario so that scientific material can be preserved for future generations.

## Introduction

Historical data are very important for evaluating long term ecological or evolutionary patterns. For example, identifying and retrieving historical data/specimens may be important in helping to reduce gaps in our knowledge regarding the past distribution of species. However, the scientific community is not doing enough to preserve raw data [1].

Modern bioacoustics, the study of animal sounds, is a relatively recent area of research. It includes all aspects related to animal sounds (including their production, transmission and reception). Sound recordings are central to the study of bioacoustics and capture and preserve acoustic events and their associated metadata [2]. Following a slow development during the first half of the 20^th^ century, mostly due to technical limitations, bioacoustics flourished towards the end of the century as rapid technological developments improved the quality of sound recordings [3]. A large body of sound recordings has accumulated and is used to investigate many diverse aspects of animal life [4], particularly the detailed study of species sound signatures [5], [6]. Sound recordings represent primary source of information (such as scientific specimens) with important scientific value and provide records of a given species in a given place at a given time. They include useful data on species that can be revisited to verify past results and to test new hypotheses.

Preservation of recording sets is critical to bioacoustics because most recordings are neither catalogued nor deposited in organized sound archives or other institutions dedicated to the long-term curation of such data, and are therefore at risk of being lost. Especially at risk are sets of recordings that are kept only by the original recordist and thus at imminent risk of loss as recordists change career, retire, or die. Older sets of recordings are at particular risk of being misplaced, of degradation of the recording storage medium, and of the technology required to play them being discontinued [7]. Many sets of recordings remain in analogue media such as tape cassettes, reel-to-reel tapes or CDs/DVDs with an unknown life expectancy. In the case of low quality CD or discontinued technology, such as DAT (digital audio tape, Sony corp., Japan) or MINIDISC (magneto-optical disc system, Sony corp., Japan) the life expectancy of the format can be as short as 5–20 years [8], [9].

This study investigated the bioacoustics legacy in Portugal. It identified sets of natural sound recordings made in Portugal and determines the rate of loss and recoverability of these resources. The study was based on information collected during a broad historical study of bioacoustics [10], which surveyed all the known recordists making natural sound recordings in Portugal. The first step in the process of retrieving animal sound recordings not preserved in archives was to identify the existing recording sets, and then to estimate their rate of loss and assess their recoverability and risk of total loss. Finally, the importance of the recoverable recording sets was evaluated on the basis of geographic, taxonomic and temporal criteria; these are key factors in establishing a retrieval and preservation strategy.

## Methods

### Research approach

**Figure 1:**
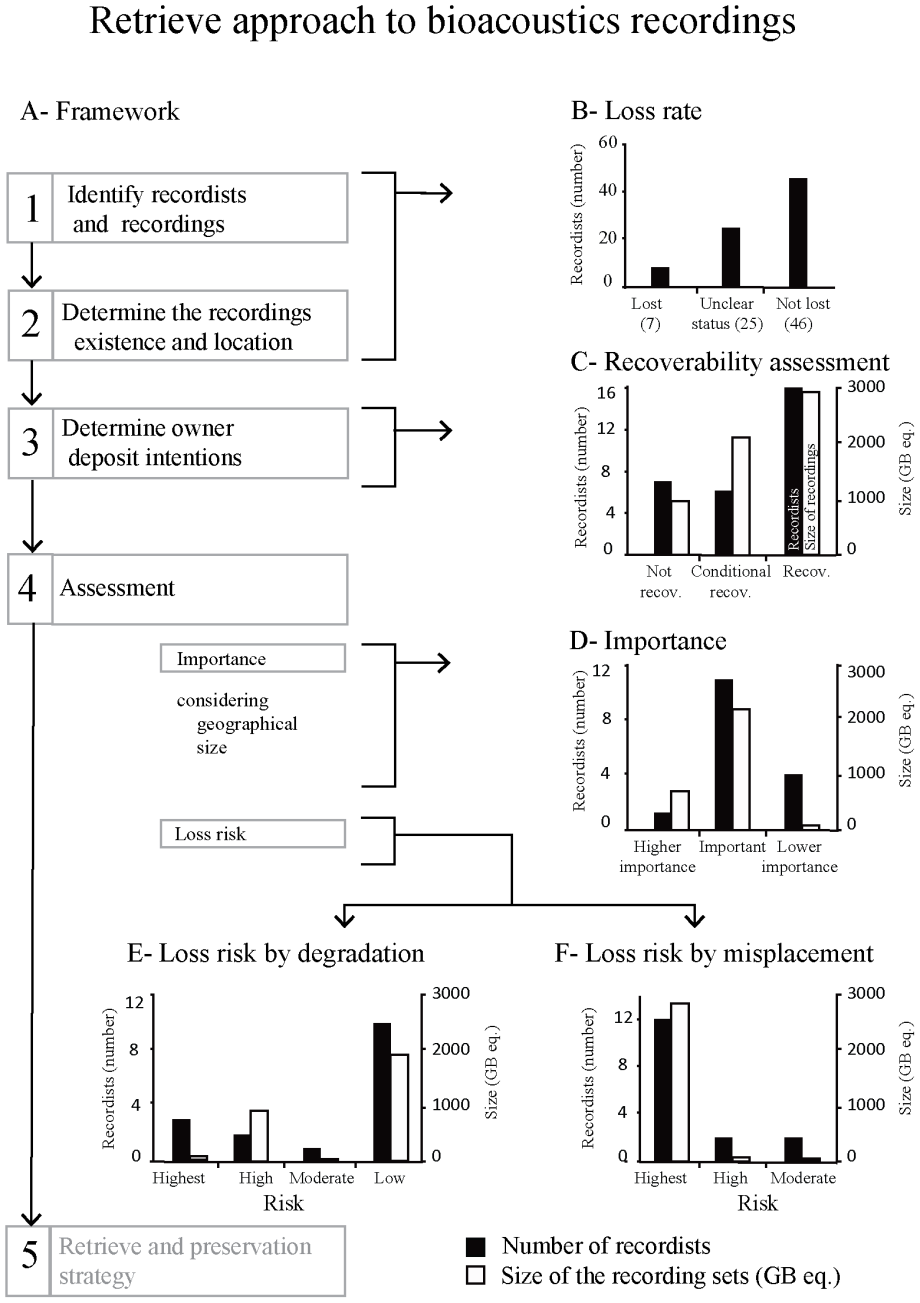
Framework to retrieve non-preserved bioacoustics recording sets made in Portugal using a history of biology information and results obtain in each step. A- Framework; B- Loss rate (78 recordists); C- Recoverability assessment (29 recordists, 6116 GB eq.); D- importance; E- loss risk by degradation and F- loss risk by misplacement (for D, E, F 16 recordists, 2975 GB eq., for details see methods section).

This study followed a retrieval framework based on four steps prior to the development of a retrieval and preservation strategy [2]: 1) the recordists were identified; 2) the existence and location of sets of recordings was determined; 3) the intentions of recordists regarding donation of their recordings were evaluated; and 4) sets of recordings were assessed for their importance and risk of loss (**Figure 1A**). Recordists were surveyed to collect the required information.

### Identification and survey of recordists

Recordists of natural animal sounds made in Portugal were identified by searching the Web of Science (Thompson Reuters, NY, USA) and Google Scholar (Google corp., MA, USA) for articles matching combinations of the search words “bioacoustics”, “Portugal”, “song” and “call” between January 1983 and December 2010. This list was then improved by searching online sound resources (Xeno-Canto (http://www.xeno-canto.org/) and nature recordists' mailing list archives (http://bioacoustics.cse.unsw.edu.au/archives/html/naturerecordists/and www.freesound.org/) for references to Portugal. The searches were performed in the month before each of the two contact periods (i.e. in August 2010 and in September 2011). Data collection from papers included the name of the corresponding author and the corresponding author email address. If the email is not working or no email information was found, we actively searched online for a recent or current email address. Moreover, the list was complimented with information gathered from the survey of recordists.

In the first contact period an email (either in Portuguese or English; see Text S1 - English version) was sent out to the recordists identified (October-November 2010; Table S1). Reminders were sent out to unanswered emails a month later. A second contact was made in November 2011 to newly identified recordists, to those who had still not answered, and also to those who had acknowledged receipt of the questionnaire but not returned it. In both contact periods the questionnaire (either in Portuguese or English for questionnaire details see the Survey Form in Text S2- English version) was sent out by email only to recordists who replied indicating their willingness to participate in the survey (consentment), or alternatively an interview appointment was made (the interview followed exactly the same questions drawn on the questionnaire). Data collection was closed in December 2011. No special approval was obtained for interviewing researchers since national authorities, funding agency and home institutions, did not require it and no such committee existed at the time of the project approval. Similar studies have not need such approval.

The survey (by interview or questionnaire) set out to collect information on each recordists' set of recordings, to determine their rate of loss, to assess their recoverability, and to assess the importance of the recordings and the risk of their being lost. Among other questions, recordists were asked to list their recording projects and species.

Recording sets identified from each recordist were logged as being: reel-to-reel tapes; Minidisks; cassette tapes; DAT tapes; CD and DVD; or HD including hard drives and other digital platforms (when a set of recordings was in different supporting media it was classified in the oldest format, overestimating loss risk by degradation). The size of each set of recordings in non-digital media was estimated by converting them to a digital measure in gigabytes equivalent (GB eq.). To calculate the size of a recording set in a specific medium the number of individual recordings (n) was multiplied by a digital conversion factor (a) and by its duration in minutes (t) (size = n×a×t). The digital conversion factor (a) used was 0.0101 GB/minute assuming a sampling frequency of 44 kHz at a 16 bits rate. Tapes (reel-to-reel tapes; Minidisks; cassette tapes; DAT tapes) were assumed to be 60 minutes long (equivalent to 0.606 GB). CDs were assumed to hold 0.600 GB and DVDs 4 GB of recordings. The state of preservation of sets of recordings was classified as; 1) preserved (if deposited in a sound archive or equivalent); 2) unpreserved (if not deposited), and the deposit intentions of the recordist noted. Moreover, the possession of the recording sets was determined and classified as: with the author; in a scientific institution; or in a web-based repository (e.g. www.xeno-canto.org). When a set of recordings was in different state of preservation it was classified in the best preservation class, overestimating the preservation status).

### Loss rate and recoverability

From the recordists initially identified, the rate of loss was estimated based on the email reply during our two contact periods. Recordings were classified as: 1) lost, if the recordists can no longer be contacted because of broken emails or no email address was found (i.e. the total number of uncontactable recordists); 2), unclear status, if no reply was received from the recordists or if a reply was received but the recordist did not return the questionnaire (i.e. the number of recordists contacted but who did not provide information); and 3) not lost, if the recordist returned the completed questionnaire (i.e. the number of recordists who provided information).

Recoverability of unpreserved sets of recordings was assessed using the information regarding the deposit intentions of their recordists. In this study we consider a recording “recoverable” if it was still possible to deposit it in an Institution dedicated to long-term data-curation. Unpreserved sets of recordings were classified in one of three classes: recoverable, if they were held by recordists willing to deposit them; conditionally recoverable, if they were held by recordists willing to deposit them subject to specific demands; and not recoverable, if held by recordists not willing to deposit them.

### Importance and risk of loss

Recoverable sets of recordings were assessed for their importance and risk of loss. The importance assessment for each recording set was evaluated according to the following criteria: the geographic coverage and the size of the recording set. The following scores were used for geographic coverage: 1) national; 2) large region; 3) small region; and 4) local; and for size 1) more than 500 GB; 2) between 100 and 499 GB; 3) between 10 and 99 GB; and 4) less than 9 GB. Importance was assessed as the sum of these scores and classified as follows: 1) higher importance (2–3 points); 2) important (4–6 points): and 3) lower importance (7–8 points).

Sets of records were further assessed according to the risk of their being lost by degradation and changes in technology, and by the risk of their being misplaced, and were classified on a scale of decreasing risk from 1 to 4. The degradation and technological obsolesce risk was scored using the scale: 1) open-reel tapes and minidisks; 2) Tapes (DAT and cassette); 3) DVD and CD; and 4) HD, representing decreasing risk of loss. The risk of their being misplaced was assessed for each set of recordings and classified in decreasing risk according to who held the original recordings as follows: 1) held by the recordist; 2) held in scientific institutions; and 3) held in web-based repository (e.g. www.xeno-canto.org). The higher risk classification of “scientific institutions” is related to the absence of enforcement policies for data preservation and specialized personnel for data-curation.

## Results

### Rate of loss rate and recoverability

The study identified a total of 78 recordists of natural animal sounds, from these 31 were identified in articles; 35 suggested by other recordists (i.e. within their survey), 8 in Xenocanto and 4 in mailing lists. In total we were able to survey 46 recordists (not lost, **Figure 1B**). The remaining recordists were either no longer contactable (9%; recordist's set of recordings classified as lost, **Figure 1B**), or did not answer our contact or did not provide any information (32%; unclear status, **Figure 1B**). On the other hand, five of the 46 recordists surveyed (11%) had already deposited their sets of recordings in archives for preservation (162 GB eq.).

Of the surveyed recordists with unpreserved sets of recordings, only 29 (6116 GB eq.) clarified their intentions to deposit recordings or not, allowing an assessment of the recoverability of sets of recordings. Most of the recordists with undeposited sets of recordings were willing to deposit them (recoverable recording sets thus representing 49% of the recording sets), or would deposit their recordings under certain conditions (conditionally recoverable, 35% of the known sets of recordings). Almost a quarter of the recordists declared no intention to deposit their recorded material for preservation (16% of the known sets of recordings, **Figure 1C**).

### Importance and risk of loss assessment

The importance of the recoverable sets of recording was calculated by combining information about geographical coverage and size. Four of the recordists recording sets were classified as of lower importance, however, representing only circa 3% of the recording size (**Figure 1D**). One set of recordings was classified as of high importance (corresponding to circa 24% of the size of the sets of recordings, **Figure 1D**) And 11 sets of recordings were classified as important,(representing 74% of the size of the sets of recordings, **Figure 1D** **).**

Regarding the risk of degradation and technological obsolesce, the majority of the sets of recordings were classified as being at low risk (65% size of recording sets, **Figure 1E**), since most recordists currently use digital media storage systems, including hard-drives or solid-state disks. However, some recordists still hold recording sets on media such as cassette tapes, DAT, CDs or DVDs, which were classified as being at higher risk (3% size of the recording sets; **Figure 1E**).

The study of risk of loss due to recordings being misplaced revealed a different picture with 75% of the recordists holding recording sets classified as at highest risk, corresponding to 96% of the total size total sets of recordings (**Figure 1F**). This is due to the fact that most of the recording sets were solely kept by their recordists. The remaining sets of recordings were either deposited in institutions (high risk) or web-based collections (moderate risk; **Figure 1F**).

**Figure 2:**
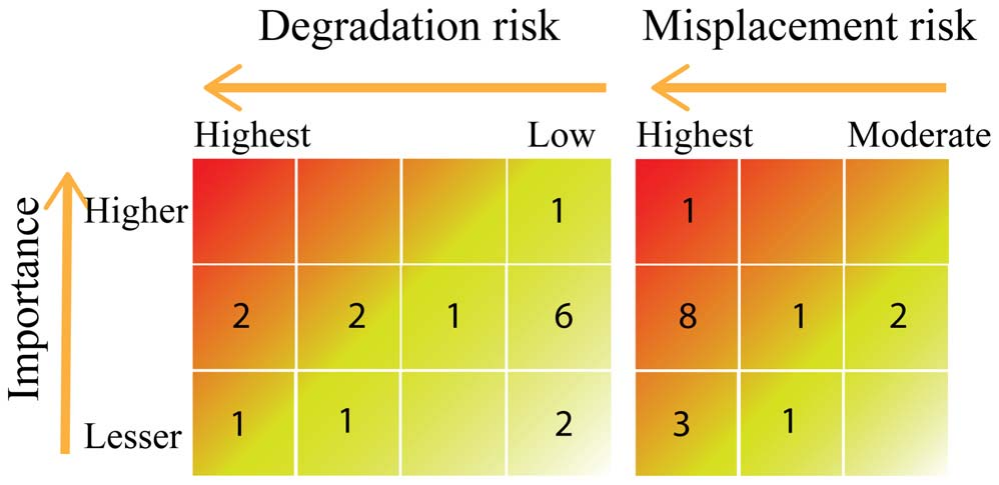
Degradation and misplacement risk of recording sets of according to importance class. Colour gradient depicting preservation priority (harmer colours- higher priority).

Importantly, the sets of recordings classified as of higher importance are not at high risk of degradation but overall they do show a moderate to high risk of being misplaced. (**Figure 2**).

## Discussion

This study discovered highly important sets of recordings at risk of being lost and these should be the subject of a preservation process to safeguard them as part of our scientific legacy. Among them are the first Portuguese sets of research recordings of animals (in the late 1970's), including recordings used to describe new species and large recording sets documenting natural areas.

Modern bioacoustics is a recent area of research [11] particularly in Portugal (Marques et al. 2011) although much of the recorded material has been classified as unclear status (32% of the recording sets, i.e. corresponding to unanswered emails) and 9% as lost. The high level of recordings with no information is due to recordists who could not be contacted and who did not answer our emails, possibly because they have moved to other institutions and/or retired. Many may have moved to different areas of study [12], [13] and related email decay in the lost recordings [12]. This difficult in collecting information about recording sets is in line with the general difficulty of locating research data as time goes by [1]. This difficulty enhance the urgent need to plan a preservation strategy for recorded material. The implementation of very strict but easy rules (e.g. an obligation to deposit recordings used in describing new species) will prevent future loss of sets of recordings and the information associated with them. It should be emphasised that the documentation associated with a recording is critical to maximise its scientific value [5], [14]. The implementation of policies on data access for publicly funded research at the national and international level [15] promises to contribute to reducing the risk of data loss in the future.

Importantly, the recoverability assessment revealed a high percentage of recording sets that could be recovered, with more than 55% of the recordists expressing their intentions to deposit their recordings in public sound archives. Information on the recoverability of recordings is vital to the elaboration of a preservation plan, since it quantifies the pool of unpreserved recording sets available. This study for Portugal estimated that there is approximately 3000 GB eq. of recoverable recording sets yet to be archived – more than that currently deposited in the Portuguese natural sound archive (c. 2200 GB).

However, the task of preserving sets of recordings presents a significant challenge [16] and it is essential to first assess their importance in order to prioritise the retrieval and archiving of the most valuable material [8]. In the present study, 75% of the recording sets are classified as important or of higher importance. The recordings in the higher importance class (6%) comprise a large recording set of a national scale project. We should point out that the assessment of importance is always subjective and dependent on a specific set of elements that may balance historic value, geographic importance taxonomic coverage and the quality of the associated documentation.

As well as quantifying the importance of a recording set, the risk of loss of recordings must also be assessed, especially if planning for retrieval or preservation of recordings. The risk of loss risk by degradation or technological obsolescence presents a very real threat to media supported material such as sound recordings [8], [16]. This risk arises from the uncertainty surrounding the life expectancy of support for media such as CDs or tapes as formats and equipment to play them, such as reel-to-reel systems, optical disks or DAT, becomes obsolete [9]. Fortunately, in our study the risk of loss through degradation or technological obsolescence is not a major problem and the bulk of recording sets are classified as low risk (i.e. sets of recording are either registered or stored in a digital format). The recording sets classified as at highest risk (Figure 1E) are held in support media that have been discontinued and are very difficult to access. Urgent attention needs to be directed towards those at high risk of loss by their being misplaced with 75% of the recording sets classified in the highest risk class, i.e. they are stored solely with their authors. This poses a significant threat since, as this study shows, the loss risk of research data is high and increases rapidly with time [1]. Our results should alert recordists to the need to safeguard their sets of recordings by depositing them in public sound archives. Furthermore, our findings emphasise the need for future research projects to incorporate plans to preserve important recording sets after the project has ended. Sets of recordings made in remote locations, of rare and endangered species, or which describe new species have great scientific value and need to be preserved. Plans to archive recordings should incorporate a strategy for their preservation that guaranties their deposit and preservation as well as including proper documentation according to long-term data-curation standards.

## Supporting Information

Table S1 Recordist information collected. Contact (1 = surveyed, 0 =  not contacted or contact not surveyed; lost- broken email or no email found); deposit intention ((1 = yes, 0 = no, 3 = cond, 4 = already deposit, na = not answered); size (na = not answered); Importance (higher importance, important, lower importance); Loss risk by degradation and tecnological discontinuity (4 low – 1 highest); and Loss risk by misplacement (3 moderate - 1 highest).
(PDF)

Text S1 Email text used to contacted recordists. Initial contact email and survey sending, English versions.
(PDF)

Text S2 Survey form sent to recordists structured in three sections. A – The researcher/recordist (questions 1 to 15), B – Equipment (questions 16 to 18), C – Recordings (questions 19 to 26) and D – Sound Archives and others (questions 26 to 31).
(PDF)
