# Supplementary material for: From the Past to the Future: Natural Sound Recordings and the Preservation of the Bioacoustics Legacy in Portugal
Source: PLoS One. 2014 Dec 4;9(12):e114303. doi: 10.1371/journal.pone.0114303 (PMC4256408; doi:10.1371/journal.pone.0114303)
Supplement: Table S1 — Recordist information collected. Contact (1 = surveyed, 0 = not contacted or contact not surveyed; lost- broken email or no email found); deposit intention ((1 = yes, 0 = no, 3 = cond, 4 = already deposit, na = not answered); size (na = not answered); Importance (higher importance, important, lower importance); Loss risk by degradation and tecnological discontinuity (4 low – 1 highest); and Loss risk by misplacement (3 moderate - 1 highest). (PDF) [file pone.0114303.s001.pdf]

Supplement material

Table S1 - Recordist information used in “From the past to the future: natural sound recordings and the preservation of the bioacoustics legacy in Portugal” Paulo A. M. Marques, Daniel M. Magalhães, Susana F. Pereira and Paulo E. Jorge.

Contact (1=surveyed, 0= not contacted or contact not surveyed; lost- broken email or no email found); deposit intention ((1=yes, 0=no, 3=cond, 4=already deposit, na=not answered); size (na=not answered); Importance (higher importance, important, lower importance); Loss risk by degradation and tecnological discontinuity (4 low - 1 highest); and Loss risk by misplacement (3 moderate - 1 highest).

Recordists	Source recordist identification	Contact period	Contact	Deposit intention	size(GB)	Importance	Loss risk degradation	Loss risk misplacement
1	Xenocanto	2	0					
2	Other recordists	2	0					
3	Other recordists	2	1	3	12.12			
4	Articles	2	1	1	700	Higher imp.	4	1
5	Other recordists	2	1	1	40	Lower imp.	4	2
6	Other recordists	2	1	na	5			
7	Articles	2	0					
8	Xenocanto	2	1	3	40			
9	Other recordists	1	0					
10	Other recordists	1	1	1	40	Important	3	2
11	Other recordists	1	0					
12	Articles		Lost					
13	Other recordists	1	Lost					
14	Articles	1	1	0	12.27			
15	Articles	1	1	4	0			
16	Other recordists	2	0					
17	Other recordists	1	1	na	0			
18	Articles	1	1	0	103.03			
19	Xenocanto	1	1	1	40	Important	4	3
20	Xenocanto	2	1	na	na			
21	Articles	1	1	1	40	Important	4	1
22	Other recordists	1	1	na	0			
23	Other recordists	1	0					
24	Other recordists	1	1	4	1.21			
25	Other recordists	1	0					
26	Other recordists	1	1	na	0			
27	mailing list	1	1	1	5	Important.	4	1
28	Articles	1	1	na	0			
29	Other recordists	2	0					
30	mailing list	1	0					
31	Other recordists	1	0					

32	Articles	2	0					
33	Other recordists	1	0					
34	Other recordists	1	1	4	100			
35	Articles	1	1	0	25.24			
36	mailing list	2	1	1	100	Important	4	1
37	Other recordists	1	1	0	na			
38	Other recordists	1	1	1	5	lower imp.	4	1
39	Articles		Lost					
40	Articles	1	1	1	35.3	Important	1	1
41	Articles	1	1	1	1000	Important	4	1
42	Other recordists		Lost					
43	Other recordists	1	1	0	840.6			
44	Articles	1	1	1	46	Important	1	1
45	Other recordists	1	1	na	0			
46	Other recordists	1	1	1	29.09	Lower imp.	2	1
47	Other recordists	1	1	na	0			
48	Other recordists	2	0					
49	Articles	2	0					
50	Articles	1	0					
51	Articles	2	Lost					
52	Other recordists	1	0					
53	Articles	2	1	3	1000			
54	Other recordists	1	1	0	2			
55	Xenocanto	2	0					
56	Articles	2	0					
57	Articles	1	1	3	36.36			
58	Articles	1	1	1	130.91	Important	2	1
59	Xenocanto	1	0					
60	Other recordists	1	1	0	3.64			
61	Articles	1	1	1	760.6	Important	2	1
62	Articles	1	1	3	1007.3			
63	Articles	1	1	na	0			
64	Articles	2	1	4	36.36			
65	Articles	1	0					
66	Articles	1	0					
67	Other recordists	2	0					
68	Other recordists	1	1	4	24.24			
69	Xenocanto	1	1	1	1.21	Important	4	3
70	Other recordists	1	Lost					
71	Articles	1	Lost					
72	Other recordists	1	1	na	0			
73	Other recordists	1	1	na	40			
74	Xenocanto	1	0					
75	Articles	2	1	na	0			
76	Articles	1	0					
77	mailing list	1	1	1	2	Lower imp.	1	1

78	Articles	2	1	3	58.18
