# Supplementary material for: From the Past to the Future: Natural Sound Recordings and the Preservation of the Bioacoustics Legacy in Portugal
Source: PLoS One. 2014 Dec 4;9(12):e114303. doi: 10.1371/journal.pone.0114303 (PMC4256408; doi:10.1371/journal.pone.0114303)
Supplement: Text S2 — Survey form sent to recordists structured in three sections. A – The researcher/recordist (questions 1 to 15), B – Equipment (questions 16 to 18), C – Recordings (questions 19 to 26) and D – Sound Archives and others (questions 26 to 31). (PDF) [file pone.0114303.s003.pdf]

Survey

In the context of the project “Natural soundscapes in a changing world. Preserving the past and building the memories of the future” we are carrying out a study that intends to gather information on the history of Bioacoustics. Our objectives are: identify the researchers, compile information about the recordings and characterize the technical work conditions. The study is focused in the knowledge area and not in the researchers.

In the end of the project the results of the study will be sent to the interviewed researchers.

Information sheet:

Nome:

Date:

Place:

Bachelor/Year/Institution:

Degree/Year/Institution

Address/contact number of interviewee

A – The researcher/recordist

1 – How was the interest in bioacoustics born?

2 – When did you first came into contact with bioacoustics?

How?

Congress or conference ◯

Colleagues ◯

Publication ◯

Other ◯ Which?

3 – What was your background knowledge?

4 – In which year did you start working in bioacoustic?

Was that first recording made in Lab or in nature

5 – What was the geographical location?

6 – Can you write a brief summary of that first project. (Please mention the researcher in charge, project name, research areas and funding)

7 – In which stage of your academic career were you?

8 – Which equipment did you use at the time?

9 – Did you start as an isolated researcher or in a group?

Isolated ◯ In a group ◯ Which?

10 – About your research lines at the time:

10.1 – In which species or group of animals?

10.2 – Scientific areas and main questions?

10.3 – Geographical location?

11 – About your present research lines:

11.1 – In which species or group of animals?

11.2 – Scientific areas and main questions?

11.3 – Geographical location?

12 – To which research group are you connected to?

13 – Please mention partners, international partnerships and groups that you worked with in bioacoustics.

14 – Please estimate, in percentages, how much of the whole of your scientific activity was dedicated to bioacoustics. Also, name and estimate the other major areas of research you dedicated yourself to.

Bioacoustics: % %

% %

15 – Have you published any kind of work about bioacoustics?

Yes ◯ No ◯

If yes, would it be possible for us to access it?

Yes ◯ No ◯

If you answered yes, we shall contact you for more information.

B – Equipment

16 – How have you acquired your initial equipment?

17 – Where could it be found?

18 – What other equipments (brand and model) have you used since (including the present one)?

C – Recordings

19 – Do you have recordings that belong to you? Yes ◯ No◯

20 – Describe project, species, date and location of your owned recordings.

21 – In which type of recording hardware are they?

HD ☐ CF ☐ CD/Cassette ☐ Other ☐ Which?

22 – Can you estimate the size of your collection of sounds?

Up to 5 Gb ◯ Between 5 and 100 Gb ◯ More then 100Gb ◯

Other units (e.g.: Nº of cassettes)

23 – Is your collection saved in some sort of backup? Yes ◯ No ◯

In which recording hardware is the backup?

HD ☐ CF ☐ CD/Cassette ☐ Other ☐ Which?

24 – Have you made sound records deposits in any museum or archives?

Yes ◯ No ◯

25 – Is the recording's information recorded together with the sound? Yes ◯

No ◯

26 – Do you find these types of recording as items with historical value, that should, like other scientific sample, be safekept as national scientific inheritance?

Yes ◯ No ◯

D – Sound Archives

27 – Would you consider deposit/donate your sound recordings? Yes ◯ No ◯

28 – Would you consider doing it in the Portuguese National Museum of Natural History? Yes ◯ No ◯

If you answered yes, we shall be in touch in a near future.

Other

29 – Do you know of anyone that, inside or outside the scientific community, made animal sound recordings in Portugal?

30 – Who, in your opinion, where the pioneers of bioacoustics in Portugal?

31 – As an experienced researcher in bioacoustic's research line, do you feel that it has unexplored potentials in a specific scientific area?

We greatly appreciate the time you spent filling our survey. Please mail it back to us.

We would be happy to answer any question you might have. We are available in these addresses: biofrodo@hotmail.com and susamaria@netcabo.pt

If you are interested in our project, you will be able to find some more information on it here: http://sites.google.com/site/paisagemacusticapt/paisagem-acustica-en
