# Supplementary material for: From the Past to the Future: Natural Sound Recordings and the Preservation of the Bioacoustics Legacy in Portugal
Source: PLoS One. 2014 Dec 4;9(12):e114303. doi: 10.1371/journal.pone.0114303 (PMC4256408; doi:10.1371/journal.pone.0114303)
Supplement: Text S1 — Email text used to contacted recordists. Initial contact email and survey sending, English versions. (PDF) [file pone.0114303.s002.pdf]

initial contact email

Dear Dr XXXXXX
In the context of the Project "Natural Landscapes in a changing World. Preserving the past and building the memories of the future", we are studying the history of bioacoustics in Portugal and we would like to have your input in our study.

In this study we intend to record the evolution of bioacoustics in Portugal as a case-study and as such, we are enquiring researcher that have dedicated themselves to the study of animal sounds.

We would appreciate if you could find the time to answer our inquiry. The final results of the study will be presented to all that answered to it.

If you agree in taking part of the study, we shall send you the inquiry. A total of 32 question will be presented on it, many of them to be easily answered.

If you have any question, please contact us.
If you wish to choose, versions in portuguese and english are available.
We thank you for your attention on this mather.

Best regards,

survey sending

Dear Dr XXXXXXX,

We are glad to know of your availability to answer our survey.
The file is in attachment. You should be able to fill the requested information by clicking in front or right under the questions.
If you should experience any problems with the file, we can supply the .doc version.

If possible, we would like to get your answer by the end of November.
Any question at all, don't hesitate to contact us.

Best regards,
